# A Compound-Centric Framework for Mapping Plant Chemical Space through Structural Scaffolds and Bioactivity Evidence

**DOI:** 10.1021/acsomega.6c04145

**Published:** 2026-06-19

**Authors:** Carlos Alexandre Carollo, Andrey Gaspar Sorrilha-Rodrigues, Mariana Calarge Nocetti, Flavio Macedo Alves, Aline Regina Hellmann Carollo

**Affiliations:** † Laboratory of Natural Products and Mass Spectrometry (LaPNEM), 54534 Federal University of Mato Grosso do Sul, 79070-900 Campo Grande, MS , Brazil; ‡ LaSMiNano - Laboratory of Micro- and Nanostructured Systems, 54534 Federal University of Mato Grosso do Sul, 79070-900 Campo Grande, MS, Brazil; § Laboratory of Botany, 54534 Federal University of Mato Grosso do Sul, 79070-900 Campo Grande, MS , Brazil

## Abstract

Open repositories have greatly expanded access to plant chemical occurrence data at scale, yet their direct use in compound prioritization remains limited by taxonomic inconsistency, structural redundancy, and uneven bioactivity annotation. Here, we present a compound-centric framework that transforms literature-curated occurrence records into interpretable, evidence-aware outputs by integrating LOTUS species–structure associations, World Flora Online taxonomic reconciliation, InChIKey-based dereplication, Murcko scaffold analysis, and conservative ChEMBL-linked bioactivity evidence. Applied to Phyllanthaceae as a case study, the workflow organizes reported chemical space across genera, resolves structure-level redundancy, and links scaffold-level novelty to family-level rarity and indexed activity records. This strategy distinguishes two complementary evidence-aware categories: lineage-restricted compounds with ChEMBL-linked activity records (STARS) and lineage-restricted compounds lacking qualifying ChEMBL activity records (HIDDEN GEMS). More broadly, the taxon-flexible design supports analyses across multiple taxonomic scales in plant lineages represented in LOTUS. It provides an auditable framework for converting heterogeneous occurrence data into transparent outputs that support evidence-aware prioritization in natural products research.

## Graphical abstract

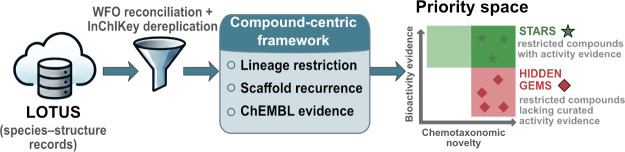

## 1 Introduction

Natural products remain a major source of bioactive compounds for drug discovery, agrochemicals, and chemical biology.^1,2^ In parallel, the discovery landscape has become increasingly data-rich through open repositories, large-scale curation efforts, omics-enabled annotation, and high-throughput analytical workflows.^3^ As a result, the central bottleneck is no longer access to chemical information alone, but the ability to convert heterogeneous chemical records into interpretable and reproducible selection workflows.

In many settings, only a small fraction of reported or detected compounds can be advanced for further investigation. This makes transparent selection strategies essential.^4^ The challenge is particularly acute for plant chemical occurrence data, where structurally redundant records, inconsistent taxonomic naming, and uneven bioactivity annotation can obscure which compounds are most suitable for focused follow-up.

Open natural product resources provide a scalable basis for addressing this challenge. LOTUS, for example, compiles referenced species-structure associations into an openly accessible resource that supports large-scale exploration of plant chemical occurrence data.^5^ However, LOTUS is primarily an occurrence-oriented resource. Using these records for compound selection requires additional downstream steps to address bias, redundancy, and evidential heterogeneity. First, occurrence data are strongly affected by taxonomic ambiguity. Synonyms, outdated names, and inconsistent organism fields can fragment records across multiple taxonomic identities. This weakens lineage-level comparisons and makes reconciliation against a standardized taxonomic backbone essential.^6^ Second, chemical records remain vulnerable to identifier redundancy, because the same structure may appear under different names or representations across sources. Without structure-based consolidation, apparent diversity may be inflated, and downstream comparisons may be difficult to interpret.

These limitations motivate a compound-centric strategy in which unique structures, rather than taxon-level metabolite lists, serve as the primary analytical unit. In this framework, the taxonomic scope is flexible. Compound selection is performed at the level of dereplicated structures. The first dimension is taxonomic restriction, which describes how narrowly a compound is represented across reported plant lineages. The second is structural organization beyond broad chemical class labels. Murcko scaffolds provide a compact representation of core ring systems and linkers, helping distinguish repeated analog series from structurally distinct frameworks.^7,8^ The third dimension is bioactivity evidence. ChEMBL provides a curated resource of standardized activity records that can serve as a conservative evidence layer for compound selection when identifier matching and record filtering are handled carefully.^9^ Together, these components support a reproducible and auditable framework for organizing reported plant chemical space.

Here, we present a reproducible compound-centric analytical framework that advances the use of plant chemical occurrence data by converting LOTUS-derived records into taxonomically normalized, structure-resolved, and evidence-stratified outputs. The workflow integrates World Flora Online (WFO) reconciliation, InChIKey-based dereplication, Murcko scaffold analysis, global family level rarity metrics, physicochemical profiling, and conservative ChEMBL-linked bioactivity evidence. By connecting these layers, the framework supports systematic data mining, visualization of lineage-associated chemical patterns, and transparent candidate stratification across plant lineages represented in LOTUS.

We demonstrate the approach in Phyllanthaceae, a chemically diverse lineage with a recently revised phylogenetic classification.^10^ This case study was used to map genus-level chemical repertoires, identify scaffold recurrence and novelty, and distinguish compounds with indexed bioactivity evidence from lineage-restricted compounds that remain poorly annotated in curated bioactivity databases.

## 2 Experimental Section

### 2.1 General Experimental Procedures

All analyses were performed in R (version 4.3.0) using a modular pipeline run from a central script (Main_Pipeline_new_v2.R). A schematic overview of the workflow and data-integration strategy is provided in Figure 1, which summarizes the progression from LOTUS species–structure occurrence records to evidence-aware compound prioritization.

**Figure 1:**
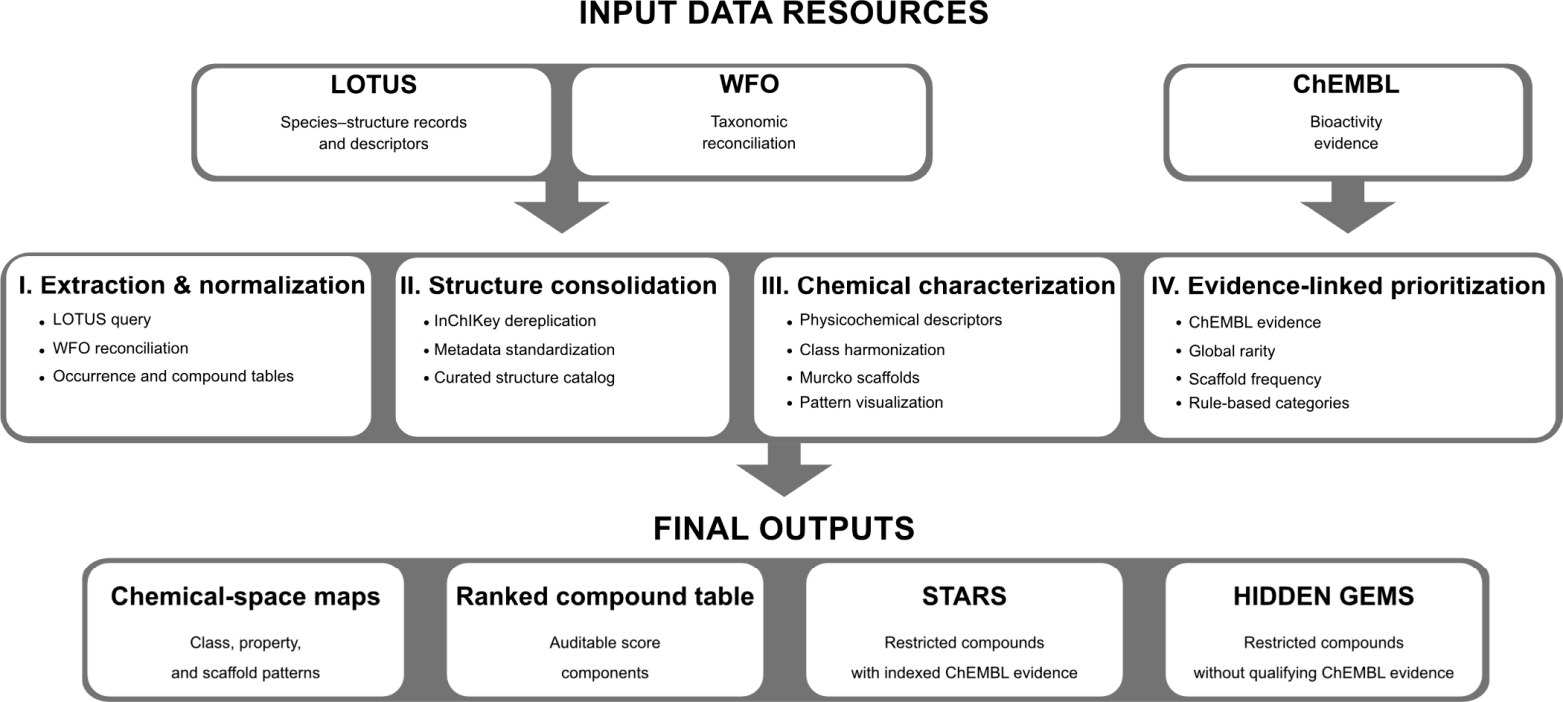
Compound-centric analytical workflow for plant chemical occurrence data. LOTUS provides species–structure records and descriptors, WFO supports taxonomic reconciliation, and ChEMBL is integrated as a parallel bioactivity-evidence layer. The workflow comprises four modules: extraction and normalization, structure consolidation, chemical characterization, and evidence-linked prioritization. Final outputs include chemical-space maps, a ranked compound table, and the STARS and HIDDEN GEMS categories.

The workflow comprises four analytical modules. Module I extracts species–compound records from LOTUS and normalizes plant names against the World Flora Online (WFO) backbone. Module II performs structure-level consolidation through InChIKey-based dereplication and generation of a curated structure catalog. Module III characterizes the reconciled compound set using physicochemical descriptors, harmonized chemical class annotation based on NPClassifier and ClassyFire, Murcko scaffold analysis, and multivariate visualization. Module IV integrates global family level rarity, ChEMBL-linked bioactivity evidence, and scaffold frequency into a rule-based prioritization procedure used to define STARS and HIDDEN GEMS.

Each module reads a shared configuration object (.cfg) containing the taxonomic scope, database connection settings, and analysis parameters. This ensures that all runs are fully scripted and noninteractive. Intermediate and final outputs were exported as Excel (.xlsx) and Parquet files in a versioned output directory, enabling independent reanalysis of each stage and a transparent audit trail across the integration layers shown in Figure 1.

### 2.2 Local Web Platform Architecture and Execution Protocol

To support reproducible execution, we implemented a documented local framework. It combines a Streamlit-based configuration interface, an R backend for modular analysis, and a local MongoDB instance containing the frozen LOTUS snapshot and derived collections.^11^

The interface writes a temporary JSON configuration file containing the taxonomic scope, run settings, and output paths. It then launches the R pipeline as a subprocess. The pipeline reads this configuration, runs the selected modules, and exports intermediate and final outputs to versioned local directories as XLSX, Parquet, and PDF files, with optional PNG previews for interface display.

This design supports dependency isolation, reproducible local execution, and traceable inspection of intermediate and final outputs.

MongoDB was run locally on port 27017 and used to store the LOTUS database and derived collections.^12^ Database persistence was maintained through the local MongoDB data directory. Initial database setup was performed through the Setup tab of the Streamlit interface, which checks for the required BSON files in the DBs/directory and restores them with mongorestore. The restored data set comprises four collections used by the workflow: unique natural products, chemical fragments, the full occurrence database, and molecular fingerprint count tables.

All components communicate through local host. The application connects to MongoDB through mongodb://127.0.0.1:27017, allowing database access without external network dependence. The Streamlit interface is available on port 8501 by default,^13^ whereas the MongoDB service remains restricted to the local machine. Output files are written directly to user-defined local directories.

During execution, user-defined parameters are written to pipeline_analysis.json, including the taxonomic scope, output directories, and database connection settings. The R script then reads this configuration, connects to MongoDB through mongolite, runs the selected modules, and exports the outputs to a versioned directory.

Tables are exported as XLSX and Parquet files, whereas figures are exported as PDF files, with optional PNG previews for interface display. Execution logs and output paths are displayed in the interface to facilitate inspection and traceability.^11^

### 2.3 LOTUS Data Extraction and Taxonomic Normalization

Primary species-compound records were obtained from a frozen LOTUS MongoDB instance (collection lotusUniqueNaturalProduct, accessed November 2025).^5^ Queries were performed with the mongolite package (v2.9.0) against the taxonomyReferenceObjects field, using either family name or genus name as the entry point (here, Phyllanthaceae and its genera). For each matched record, we retrieved the InChIKey, SMILES, IUPAC name, molecular formula, molecular weight, and available physicochemical and taxonomic metadata. Data were processed in fixed-size batches (default 5,000 records) through MongoDB aggregation pipelines using dplyr, data.table, and progress to limit memory usage.

Species names were then normalized against the World Flora Online (WFO) backbone.^14^ A local copy of the WFO taxonomic table was parsed to generate synonym-to-accepted-name mappings. Canonical species strings were generated by conversion to lowercase followed by trimming and removal of authorship and infraspecific annotations. Each LOTUS taxon was then matched to an accepted WFO name whenever possible. When a match was obtained, genus and family names were replaced by the corresponding WFO-accepted classification, and a taxonomy action flag was recorded to indicate whether the original name was confirmed or corrected. Records with unresolved names or genus-level inconsistencies were retained but explicitly labeled. The resulting long-format table (lin_enriched) contains one row per compound-species occurrence with harmonized family, genus, and species names, whereas the unique compound table (uni_enriched) was collapsed by InChIKey.

The present study focuses on a plant lineage and therefore applies WFO-based normalization. However, the workflow is modular and can also be used for other LOTUS-represented groups, including fungi and bacteria.^5^ In such cases, the WFO module should be disabled and, when required, replaced with an appropriate taxonomic backbone.

### 2.4 Chemical Descriptors and Class Ontology

Core physicochemical descriptors were obtained directly from LOTUS metadata when available. These included molecular weight (MW), xLogP, topological polar surface area (TPSA), fraction of sp3 carbons (Fsp3), hydrogen-bond donors (HBD), hydrogen-bond acceptors (HBA), and auxiliary logP estimates (AlogP, AMRA logP, and Manhold logP). Continuous variables were converted to numeric format and capped when necessary (e.g., TPSA) to limit the influence of extreme outliers.

Chemical class assignments were based on LOTUS annotations derived from established chemical classification resources, including ClassyFire and NPClassifier.^15,16^ ClassyFire provides an automated chemical taxonomy based on structural rules. NPClassifier provides a natural product-oriented structural classification system. For each structure, the primary NPClassifier superclass and the primary ClassyFire class were extracted, duplicate and multilabel entries were cleaned, and a rule-based harmonization step was applied to merge semantically overlapping categories (e.g., collapsing “Prenol lipids” into “Terpenoids” or merging related tannin subclasses). The resulting hybrid_class variable was used as an operational harmonized class descriptor for downstream visualization and comparative analyses, including heat maps, PCoA, and compound prioritization.

Murcko scaffolds were obtained from the LOTUS murko_framework field when available. For descriptive analyses, scaffold frequencies were calculated at the target taxonomic level (e.g., genus or family), yielding a local scaffold count per InChIKey (Local_Scaffold_Freq). Optional visualization of Murcko scaffold distributions was implemented in Part III using ChemmineR,^17^ but these plots were descriptive and were not required for the prioritization workflow.

### 2.5 Global Context and Rarity Metrics

To quantify global chemical rarity, the LOTUS MongoDB database was requeried for all occurrences of each InChIKey detected in uni_enriched. For each compound, the taxonomyReferenceObjects field was parsed to recover all plant families in which that compound had been reported. A dedicated helper function was used to collapse multiple taxonomic references conservatively and eliminate malformed entries. The resulting summary included (i) Global_Family_Count, defined as the number of distinct plant families in which the compound was reported, and (ii) Family_List_String, containing up to five representative family names.

Compounds were then assigned to qualitative rarity tiers as follows: “Exclusive (1 family)”, “Restricted (2–10 families)”, “Ubiquitous (>10 families)”, and “Not found/error” for failed or unresolved lookups. These tiers were used in the prioritization process and in filters designed to remove ubiquitous compounds or records with unreliable provenance.

### 2.6 Bioactivity Mining and Quality Filters (ChEMBL Layer)

Experimental bioactivity data were obtained from ChEMBL^9^ through the ChEMBL REST API (release 33, accessed November 2025).

Each InChIKey in uni_enriched was mapped to ChEMBL molecule identifiers by querying the ChEMBL molecule end point (molecule.json) in batches using the molecule_structures__standard_inchi_key__in filter. The resulting mapping table (chembl_map) was deduplicated to preserve one-to-many InChIKey–ChEMBL relationships where applicable.

For all mapped ChEMBL identifiers, bioactivity records were retrieved from the ChEMBL activities end point. Records were then filtered to retain only assays with defined standard activity types and values, namely IC_50_, EC_50_, AC_50_, GI_50_, *K*_i_, *K*_d_, and MIC, reported in nanomolar units. Records with missing units or standard values greater than 10,000 nM (10 μM) were excluded.

For each compound, the most potent retained activity was summarized as Best_Potency_nM, together with the corresponding target name, target organism, and the total number of assays (*N*_Assays). When multiple records were available for the same compound, the most potent retained activity was used as a conservative summary metric for ranking, whereas the total number of retained assays was recorded separately to preserve evidence depth.

Because assay conditions and experimental contexts may vary across ChEMBL entries, these records were treated as a curated and conservative supporting-evidence layer for candidate stratification rather than as independent experimental validation. This caution is supported by recent evidence showing that combining IC_50_ or *K*_i_ values across assays and sources can introduce substantial noise, even for the same nominal target. Stricter curation improves agreement, but it also reduces data set size.^18^

To reduce inflation of hits associated with promiscuous lipophilic binders, ligand lipophilicity efficiency (LLE) was calculated as pPotency – xLogP, where pPotency was derived from the best retained nanomolar activity value. Compounds with retained bioactivity records were flagged as activity-linked compounds, and this metric was incorporated into the prioritization procedure together with potency thresholds. Higher-confidence activity-linked compounds were defined as those with positive lipophilicity-adjusted potency values or best potency <500 nM, whereas lower-efficiency compounds with weaker potency were retained but down-weighted.

### 2.7 Prioritization and Definition of STARS and HIDDEN GEMS

The rule-based prioritization procedure integrated three transparent compound-level dimensions into a ranking score: (i) local scaffold frequency within the target lineage (Local_Scaffold_Freq), (ii) global family level rarity (Global_Family_Count), and (iii) activity evidence derived from ChEMBL records.

Local novelty contributed positively when a Murcko scaffold showed low frequency within the target taxon, defined as one to five occurrences. Global rarity contributed positively for exclusive or restricted compounds (1–3 families) and negatively for ubiquitous compounds (>10 families) or compounds not recovered in the global-context query. The activity component incorporated the presence of ChEMBL-linked bioactivity records, potency, and LLE. These components were combined into a composite PRIORITY_SCORE, which was used only for ranking within the filtered compound set.

Compounds were assigned to the STARS category when they were lineage-restricted, had ChEMBL-linked activity records, and met either LLE > 0 or Best_Potency_nM < 500 nM. Compounds were assigned to the HIDDEN GEMS category when they were lineage-restricted and had positive rarity scores but lacked qualifying ChEMBL-linked activity records. Additional categories, including “HIT (Low Efficiency)” and “BASELINE”, were used for internal diagnostics but were not central to the present study.

At this stage, a blacklist was applied to remove ubiquitous primary metabolites and common analytical or biosynthetic background compounds, including fatty acids, phytosterols, chlorophyll derivatives, and simple sugars. Compounds whose IUPAC names matched blacklist terms were automatically tagged as DISCARD (Primary Metabolite), regardless of rarity or activity status. This limited the influence of ubiquitous background metabolites on downstream prioritization.

### 2.8 Multivariate Analyses and Visualization

All visualizations and summary statistics were generated with reproducible scripts (Part III). Taxon-level ordinations were calculated by principal coordinate analysis (PCoA) using the vegan package.

Presence-absence matrices of hybrid classes per taxon were generated from lin_enriched, and Jaccard dissimilarities were calculated with vegdist. PCoA was performed with cmdscale in base R, and the first two axes (PCoA1 and PCoA2) were used for visualization.

To aid interpretation of class-level drivers, hybrid_class vectors were fitted to the ordination using envfit with 999 permutations, retaining only associations with adjusted *P* < 0.05 after multiple-testing correction. These ordination and vector-fitting procedures were used as exploratory tools to visualize chemical-class composition and identify class-level gradients, rather than as supervised classification or predictive models.

Differences among genera in molecular descriptors were assessed using nonparametric tests because these variables were not assumed to follow normal distributions. Global comparisons were performed with Kruskal–Wallis tests, followed by pairwise Wilcoxon rank-sum tests with Benjamini-Hochberg correction for multiple comparisons. Summary statistics and significant pairwise contrasts are provided in Table S3.

Heat maps of chemical class repertoires were generated with ComplexHeatmap and circlize, using chemical classes as rows and taxa as columns. Count transformations or normalizations are indicated in the corresponding figure legends. Additional density plots, box plots, prioritization matrices (novelty vs evidence), and extraction profiler panels were constructed with ggplot2, ggrepel, and scales. Plot export was handled by custom helper functions that wrote publication-quality PDF files and, when needed for interface preview, optional PNG versions.

### 2.9 Code and Data Availability

All scripts used for data extraction, curation, analysis, and prioritization of LOTUS-derived species–compound records are publicly available at the project repository: https://github.com/andreyferramentasia/SPARK-Scaffold-based-Prioritization-And-Recognition-of-Knowledge-gap-v1.3.

The repository includes the modular R pipeline, the documented local interface, configuration files, setup scripts, and user documentation required to reproduce the workflow in a local environment. External dependencies, including LOTUS database snapshots, WFO backbone tables, and ChEMBL queries, are documented together with acquisition dates, expected file formats, and run settings.

## 3 Results and Discussion

After WFO-based taxonomic reconciliation and InChIKey dereplication, we obtained a structure-resolved Phyllanthaceae data set that supports genus-level comparisons without inflation from synonymy or redundant structure representations. Figure 2 summarizes the organization of reported chemical space across Phyllanthaceae genera after taxonomic reconciliation and InChIKey-based dereplication.

**Figure 2:**
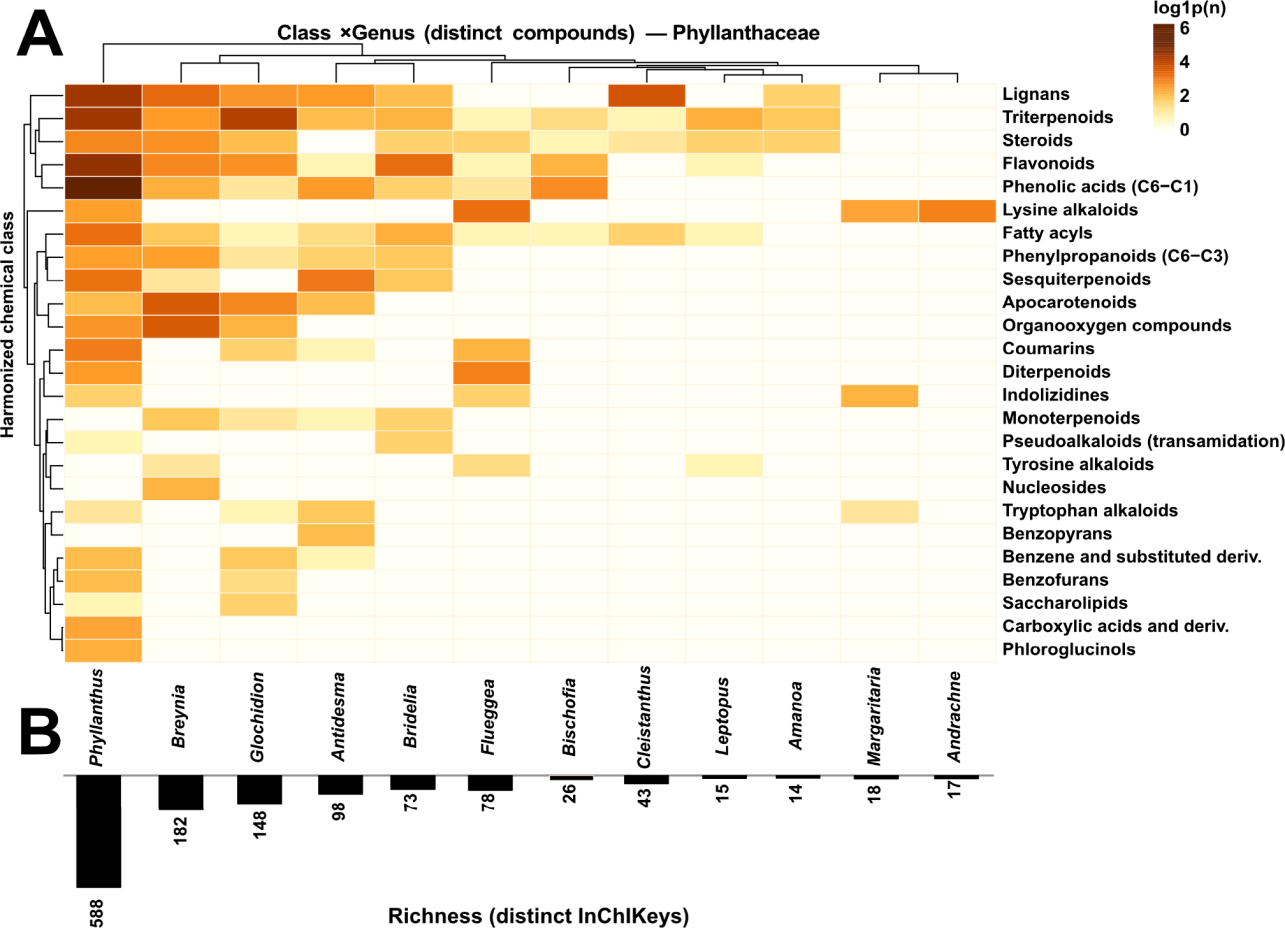
Genus-level chemical landscape of Phyllanthaceae. (A) Heatmap of distinct InChIKeys per harmonized chemical class and genus after taxonomic reconciliation and structure-level consolidation. Cell intensity represents log1p-transformed compound counts. (B) Genus-level richness based on distinct InChIKeys.

Generic circumscriptions in Phyllanthaceae, particularly within *Phyllanthus* s.l., have been repeatedly revised.^10^ Therefore, the summaries in Figure 2A,B should be interpreted as an operational view of reported chemical space anchored to the reconciled taxonomic backbone, rather than as fixed evolutionary units.

The class-by-genus heatmap reveals a heterogeneous but structured distribution of reported chemistry across Phyllanthaceae. Some genera concentrate multiple major classes, including lignans, triterpenoids, steroids, and flavonoids, whereas others display narrower class profiles. Selected examples illustrated in Figure 3 are consistent with genus-associated chemotypes described in the literature, including securinane-type alkaloids in *Margaritaria*, piperidine alkaloids in *Andrachne*, and aryltetralin lignans in *Cleistanthus*.^19–21^ More broadly, the multiclass profiles observed for genera such as *Phyllanthus* and *Breynia* are consistent with previous phytochemical reports for these taxa.^22,23^

**Figure 3:**
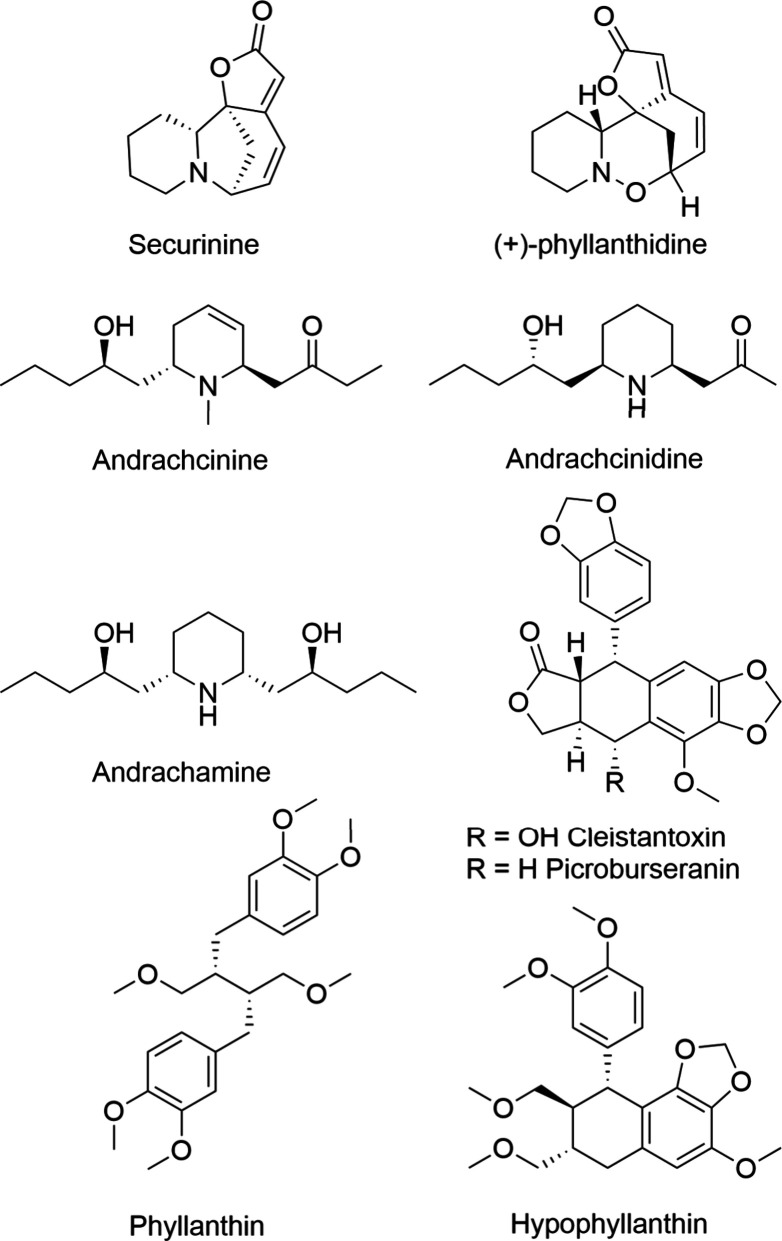
Representative Phyllanthaceae compounds discussed in the text. Shown are securinane-type alkaloids (securinine and (+)-phyllanthidine), piperidine alkaloids (andrachcinine, andrachcinidine, and andrachamine), lignans (phyllanthin and hypophyllanthin), and the arylnaphthalene lignans cleistantoxin (R = OH) and picroburseranin (R = H).

These patterns should be interpreted cautiously. Literature-curated occurrence data are shaped by uneven study effort, analytical accessibility, and reporting bias.^5^ Certain classes may be preferentially represented in the literature because they are easier to isolate, detect, or prioritize for biological evaluation. Accordingly, Figure 2A is best interpreted as a structure-resolved summary of reported genus-level repertoires rather than as a direct measure of intrinsic biosynthetic capacity.

Figure 2B further contextualizes this pattern by showing marked differences in the number of distinct InChIKeys reported per genus in the curated data set. Reported richness is uneven across Phyllanthaceae. The most represented genera were *Phyllanthus* (588), *Breynia* (182), *Glochidion* (148), *Antidesma* (98), *Flueggea* (78), and *Bridelia* (73). This pattern likely reflects both biological diversity and uneven literature coverage. Reviews of *Phyllanthus*, for example, note that some species have been investigated much more extensively than others and that many studies focused on specific metabolite classes rather than on comprehensive chemical characterization at the species level.^22^

Taken together, Figure 2 provides a standardized, structure-resolved view of the reported chemical space of Phyllanthaceae. By combining taxonomic reconciliation with InChIKey-based dereplication, the framework reduces redundancy-driven artifacts and establishes the basis for subsequent analyses of class composition, physicochemical profiles, scaffold organization, and activity-linked evidence.

To move beyond class-richness summaries, we used PCoA to examine genus-level variation in hybrid-class composition across Phyllanthaceae (Figure 4A). Although the first two axes captured modest variance (PCoA1 = 9.1%, PCoA2 = 8.4%), the ordination still showed structured separation among genera along class-composition gradients. The genera did not collapse into a diffuse family level cloud.

**Figure 4:**
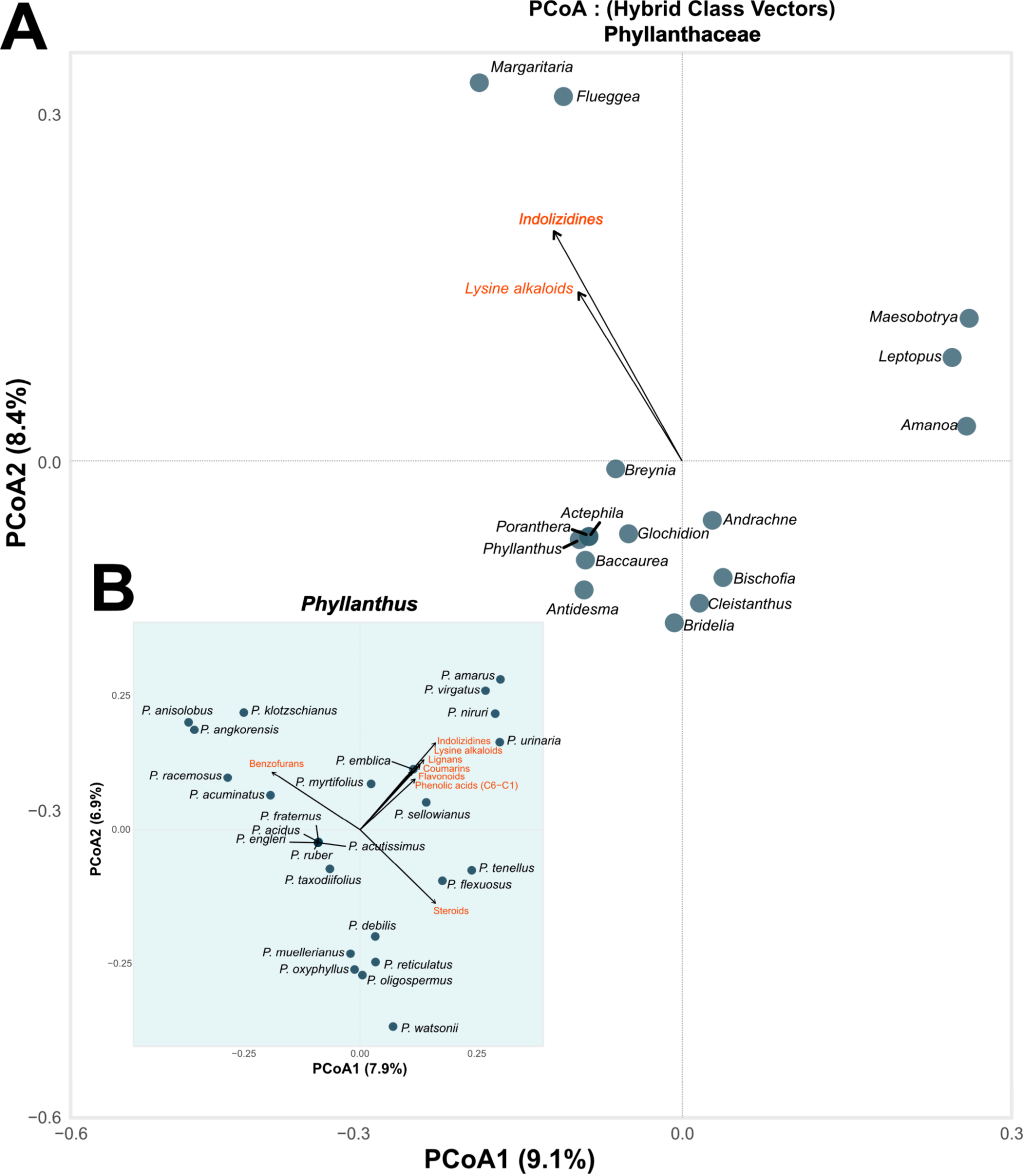
Class-composition PCoA of Phyllanthaceae and within-genus compositional variation in *Phyllanthus*. (A) Genus-level principal coordinate analysis (PCoA) based on hybrid-class composition, with distinct InChIKeys summarized into harmonized class bins as defined in the Methods. Arrows indicate fitted class vectors. (B) Species-level PCoA for *Phyllanthus*, showing within-genus variation in reported class composition and the associated fitted class vectors.

In this representation, genera are positioned according to class-abundance vectors derived from distinct InChIKeys, consistent with the compound-centric design of the workflow.^24^ Figure 4A therefore provides a compositional overview of the broad chemotypes that differentiate genera after reconciliation and dereplication.

Because genus boundaries in Phyllanthaceae have been revised repeatedly, it is reasonable to ask whether part of the observed compositional pattern is consistent with major phylogenetic groupings within tribe Phyllantheae. *Phyllanthus* has long been recognized as paraphyletic.^10^ Recent phylogeny-based revisions divided *Phyllanthus* s.l. into multiple monophyletic genera. These revisions also recognized historically nested lineages, such as *Breynia*, *Glochidion*, and *Synostemon*, as part of the broader *Phyllanthu*s complex.

Bouman et al. reported close evolutionary relationships among *Phyllanthus*, *Breynia*, and *Glochidion*.^10^ In the present analysis, the proximity of these genera in ordination space was qualitatively consistent with that relationship. Other genera were more strongly displaced in the ordination, suggesting that some genus-level chemical signatures may reflect deeper lineage differences as well as differences in sampling and literature coverage.

Likewise, the fitted alkaloid vectors point toward *Margaritaria* and related genera such as *Flueggea*, consistent with the restricted occurrence of securinane-type (“Securinega”) alkaloids in a limited subset of Phyllanthaceae genera.^25^ This example illustrates how fitted class vectors can provide chemically interpretable axes of lineage-level differentiation, without implying a strict correspondence between chemistry and phylogeny.

Among the fitted class vectors, alkaloid-associated classes contribute strongly to genus separation, with lysine alkaloids and indolizidines aligned with alkaloid-weighted genera in ordination space (Figure 4A). This pattern is consistent with the genus-associated chemotypes summarized in Figure 2, but here it emerges from multivariate class composition rather than from single-compound exemplars.^20,26^

The species-level ordination for *Phyllanthus* (Figure 4B) further reveals substantial within-genus variation in class composition. This variation was associated with different fitted class vectors, including benzofurans and steroids in addition to major phenolic and terpenoid classes. Rather than exhibiting a chemically uniform profile, *Phyllanthus* shows marked species-level compositional heterogeneity.

This pattern underscores the value of a taxon-flexible framework in which the analytical unit is the structure and the taxonomic scope can be adjusted across family-, genus-, and species-level analyses.^22^

Figure 5 summarizes genus-level differences in the physicochemical profiles of Phyllanthaceae. In panel A, a heatmap of scaled median values with hierarchical clustering on both axes shows structured variation among genera. The descriptors capture lipophilicity (xLogP), saturation and three-dimensionality (Fsp3), ring count, hydrogen-bonding capacity (HBA/HBD), polarity (TPSA), and molecular size (MW). Panel B complements this overview by showing compound-level distributions for selected descriptors, including lipophilicity (xLogP), saturation (Fsp3), hydrogen-bonding capacity (HBD), polarity (TPSA), molecular size (MW), and oxygen-to-carbon ratio (O/C ratio).

**Figure 5:**
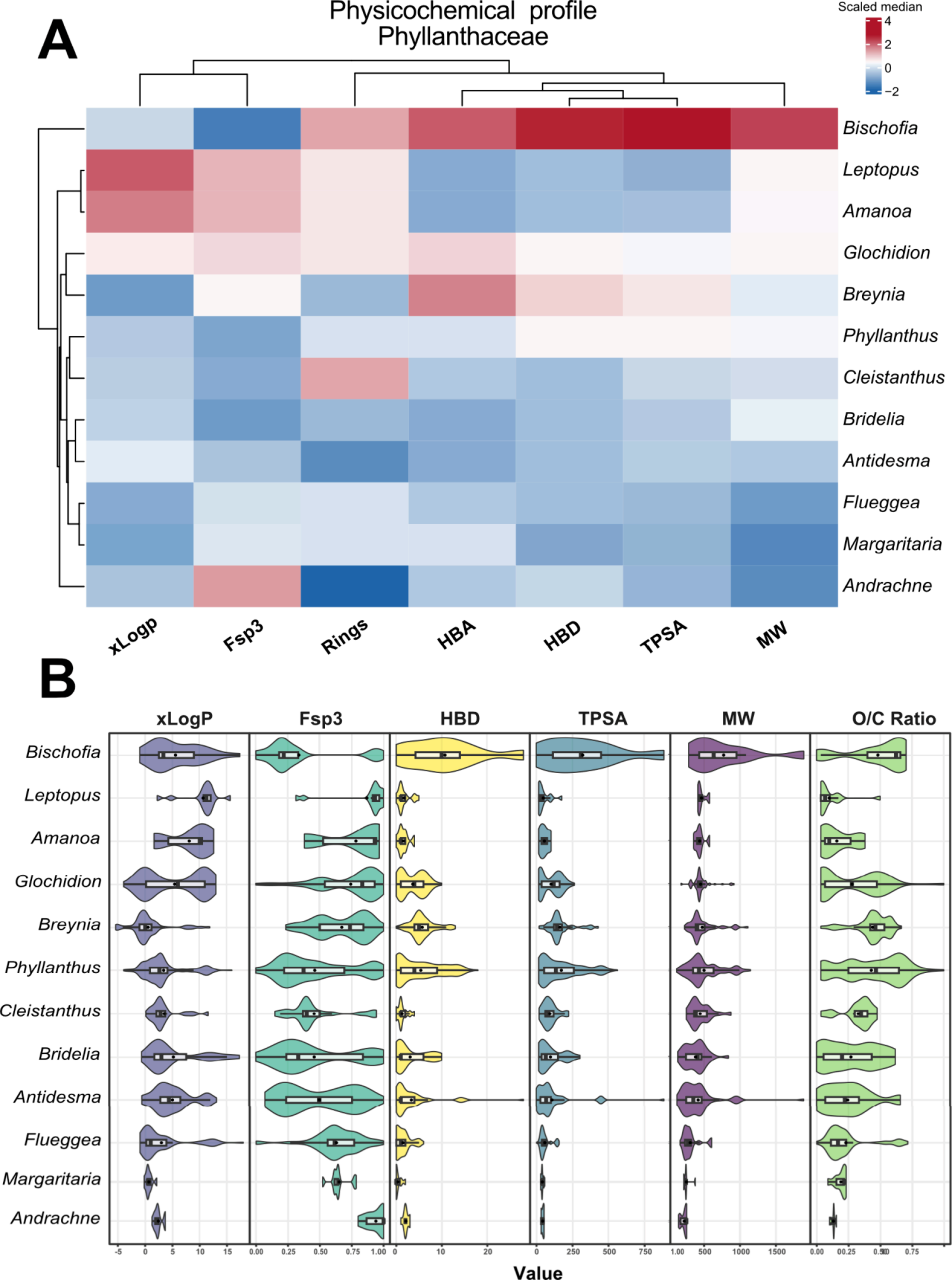
Genus-level physicochemical profiles across Phyllanthaceae. (A) Heatmap of genus-level scaled median values for key descriptors (xLogP, Fsp3, ring count, HBA, HBD, TPSA, and MW) with hierarchical clustering. (B) Violin plots showing compound-level distributions for selected descriptors (xLogP, Fsp3, HBD, TPSA, and MW) and the O/C ratio. Embedded boxplots indicate the median and interquartile range.

Across the 12 focal genera, descriptor distributions differed consistently. However, these results were interpreted primarily according to the direction, magnitude, and chemical coherence of the observed patterns rather than statistical significance alone. Global and pairwise nonparametric tests confirmed that these differences were unlikely to reflect random variation in the curated data set. The complete statistical results are provided in Table S3.

More importantly, the descriptor profiles reveal biologically and chemically meaningful regimes. Oxygen-rich and highly polar profiles were associated with elevated TPSA, HBA, HBD, and O/C values. More lipophilic and highly saturated profiles were associated with higher xLogP and Fsp3 values. These regimes provide practical information for interpreting reported chemical repertoires, anticipating extraction and detection biases, and selecting compounds for downstream evaluation.

These patterns should be interpreted with caution because the data set reflects reported chemical space and may therefore be influenced by extraction practices, analytical accessibility, and reporting bias. Differences in solvent polarity and analytical workflows across studies may further shape the observed distributions, which should be interpreted as summaries of the reported literature rather than as direct representations of the full metabolite space of each genus.

Two contrasting examples illustrate the practical meaning of the physicochemical regimes observed across genera. *Bischofia* occupies a highly polar and oxygen-rich region of the reported chemical space, with high median TPSA (310.7), strong hydrogen-bonding capacity (HBD 10; HBA 9), high molecular weight (MW 634.5), and an elevated O/C ratio (0.625; *N* = 26). This profile is consistent with reports of highly oxygenated, tannin-rich constituents in the genus.^27^ In contrast, *Leptopus* and *Amanoa* occupy a more lipophilic and highly saturated region, characterized by high median xLogP values (11.28 and 10.07, respectively), high Fsp3 values (0.93 for both), and comparatively low TPSA values (37.3 and 56.5, respectively; *N* = 15 and 14), in agreement with reports of steroid- and triterpenoid-rich chemistry in these taxa.^28,29^

These differences are not only statistically supported but also chemically informative. Polar, oxygen-rich profiles may favor different extraction, fractionation, detection, and assay conditions than lipophilic, highly saturated profiles. Thus, genus-level physicochemical regimes provide practical guidance for interpreting reported chemical repertoires and for designing downstream workflows tailored to different regions of plant chemical space. In this context, high Fsp3 values may be relevant to candidate stratification when interpreted together with scaffold structure and activity evidence, because they reflect increased saturation and three-dimensionality in the reported compound set.^30^

Between these extremes, several genera occupy intermediate profiles. For example, *Breynia* is relatively hydrophilic and polar (median xLogP 0.06; TPSA 145.9; HBD 5; *N* = 236), whereas *Phyllanthus*, the largest genus in the data set (*N* = 814), shows broad within-genus distributions around intermediate median values (xLogP 2.66; TPSA 136.7; MW 430.5). This broad distribution reinforces that large and chemically diverse genera should not be interpreted only by their median descriptor values, but also by the range of chemical space represented by their reported compounds.

Importantly, the stratified regimes in Figure 5 also map naturally into the broader concept of natural-product property space. Relative to typical synthetic libraries, natural products occupy distinct regions shaped by stereochemical richness, oxygenation, and complex ring systems. These features are captured here by coordinated shifts in O/C, TPSA, H-bonding capacity, and ring/Fsp3 profiles.^31^

In this context, genus-level clustering does more than summarize compositional variation. It shows that different genera occupy distinct physicochemical regions of reported chemical space. Oxygen-rich and more polar regimes are associated with higher TPSA, HBA, and HBD values, whereas more lipophilic and higher-Fsp3 regimes are associated with greater three-dimensionality and lower polar burden.

This interpretation aligns with quantitative NP-likeness concepts that formalize how combinations of molecular features differentiate natural-product-like structures from other chemical matter. It also supports the use of genus-resolved property profiles as a complementary layer to class composition when prioritizing candidates for isolation, fractionation strategy, and assay compatibility.^32^ Thus, genus-resolved physicochemical profiles show that genera differ not only in the classes reported for them, but also in the molecular property space occupied by their reported compounds.

Having established genus-level compositional structure, we next examined scaffold-level novelty using Bemis-Murcko frameworks.^8^ These frameworks reduce each compound to its core ring systems and linkers. This helps distinguish analog expansion around shared cores from structurally distinct framework patterns.^33^

Figure 6 summarizes this scaffold-based view in two complementary ways. Panel A shows the Top 15 Murcko frameworks (fragment MW ≥ 200 Da) ranked by frequency, with stacked bars indicating genus-level contributions to each framework. Panel B shows a genus-level structural innovation ratio, defined as the number of unique scaffolds divided by the number of distinct InChIKeys in each genus.

**Figure 6:**
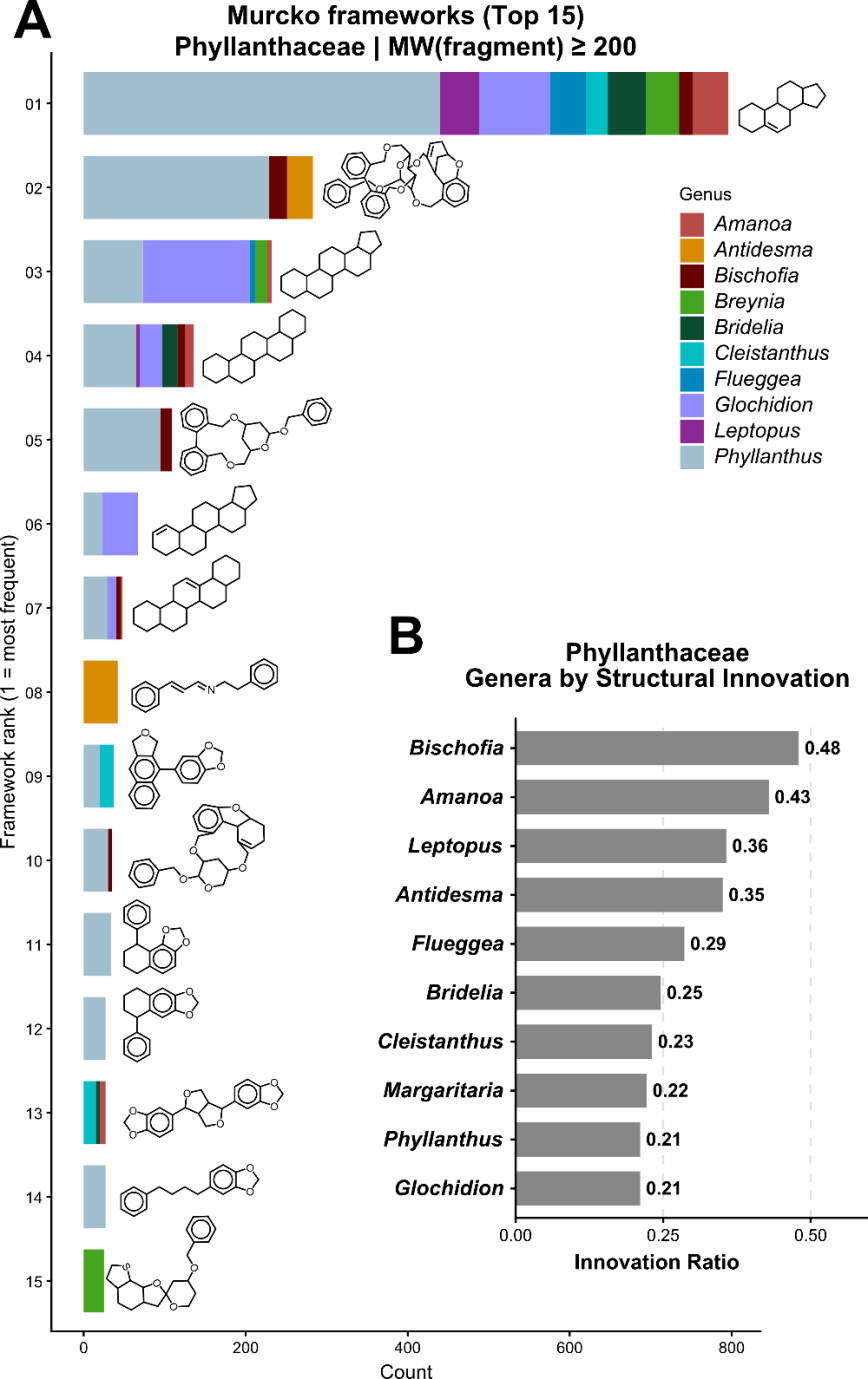
Scaffold distribution and structural innovation across Phyllanthaceae. (A) Top 15 Murcko scaffolds (fragment MW ≥ 200 Da) ranked by frequency, with stacked bars showing genus-level contributions to each scaffold and representative scaffold structures shown at right. (B) Genus-level structural innovation quantified as the Murcko scaffold-to-compound ratio (unique scaffolds/distinct InChIKeys). To aid interpretation, the underlying counts used to calculate the ratio for each genus are reported in Table S2.

This scaffold-based representation is informative in literature-curated chemical space because repeated isolation of close analogs can inflate compound counts without proportionally increasing framework diversity.^34^

Figure 6A shows that the most frequent scaffolds are unevenly distributed across Phyllanthaceae genera. The 15 most frequent frameworks included both broadly shared scaffolds and scaffolds disproportionately represented in particular genera. In some cases, a small number of genera accounted for a large share of the InChIKeys assigned to a given scaffold.

Importantly, the genus-stacked view shows that high scaffold frequency does not imply genus specificity, since several of the most frequent scaffolds receive contributions from multiple genera. This result highlights why taxonomic reconciliation and structure-level dereplication must precede any inference of taxonomic restriction. It also shows why scaffold-level novelty should be interpreted independently of raw occurrence counts.

To clarify the chemical meaning of the dominant scaffolds, we decomposed the 15 most frequent frameworks using the hybrid class annotation (NP_Class). This linked scaffold space to broader class patterns, rather than treating it as a single undifferentiated pool (Figure S1). Across these scaffolds, class composition was dominated by lignans, triterpenoids, and C6–C1 phenolic acids. Additional scaffold-specific contributions came from tannins, steroids, C6–C3 phenylpropanoids, and diarylheptanoids.

The within-scaffold enrichment profile further shows that individual frameworks are often class-biased relative to the global background. This helps explain why scaffold rarity captures information not conveyed by class counts alone. Some locally rare scaffolds correspond to distinct class-defined cores, whereas highly recurrent scaffolds often reflect expanded analog series within dominant chemotypes.

Whereas panel A identifies the dominant frameworks and their genus distribution, Figure 6B summarizes the ratio between unique scaffolds and distinct compounds in each genus. These ratios vary substantially among genera and should be interpreted with caution. High ratios in small inventories may reflect limited analog expansion around a few cores. In contrast, lower ratios in compound-rich genera may reflect sustained series expansion around recurrent scaffolds and more densely sampled analog families. Accordingly, both high and low ratios can be informative, but only when interpreted together with the underlying numbers of compounds and scaffolds in each genus. To facilitate this interpretation, the counts used to calculate the innovation ratio, including the number of distinct compounds and unique Murcko scaffolds per genus, are provided in Table S2.

Finally, scaffold rarity can also be tracked at the compound level through the Scaffold Frequency field (Table S1), which records how common each compound’s Murcko framework is within the curated lineage. In this context, locally rare scaffolds provide a complementary signal of structural novelty alongside restriction metrics, helping distinguish broader structural exploration from prolific analog series.

Figure 7 summarizes the evidence-novelty landscape of the Phyllanthaceae data set and shows how the ranking separates lineage-restricted compounds into two evidence-aware categories with distinct profiles. The STARS category comprised 22 lineage-restricted compounds reported in ≤ 3 plant families globally, including eight family exclusive compounds. Their best retained ChEMBL potencies ranged from 0.67 to 10,000 nM, and the category was enriched in lignans and coumarins. In contrast, the HIDDEN GEMS category comprised 25 family exclusive candidates without qualifying indexed ChEMBL activity records. This separation distinguishes restricted compounds with ChEMBL-linked activity records from restricted compounds lacking qualifying ChEMBL activity records under the same restriction- and scaffold-aware criteria. The complete ranked data set is provided in Table S1.

**Figure 7:**
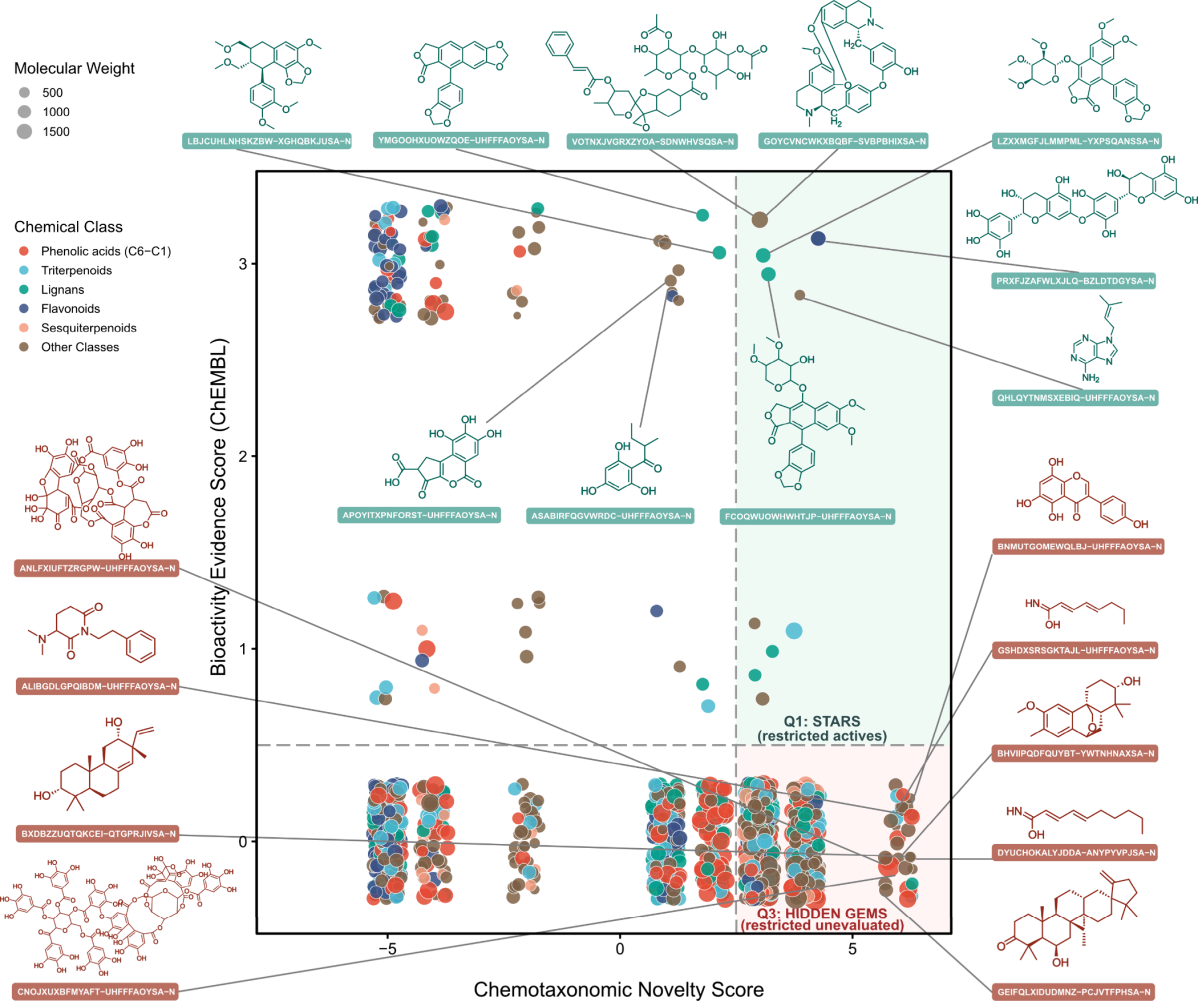
Evidence–novelty matrix distinguishing the STARS category, comprising lineage-restricted compounds with ChEMBL-linked activity records, from the HIDDEN GEMS category, comprising lineage-restricted compounds without qualifying ChEMBL activity records, in Phyllanthaceae. Each point represents a distinct InChIKey positioned by chemotaxonomic novelty score (*x*-axis) and ChEMBL-derived bioactivity evidence score (*y*-axis). Point color indicates chemical class and point size reflects molecular weight. Dashed lines denote the thresholds used to define Q1 (STARS) and Q3 (HIDDEN GEMS). Representative structures are shown for selected compounds and linked to their positions in the matrix.

The STARS quadrant (Figure 7, Q1) grouped lineage-restricted compounds linked to curated quantitative activity records. This indicates that restriction- and scaffold-aware ranking can highlight compounds that are distinctive in occurrence space and linked to indexed activity evidence. Across the Phyllanthaceae literature, several reports provide quantitative readouts across different assay contexts.

For example, dichapetalin-type triterpenoids and co-occurring lignans from *Phyllanthus acutissima* were isolated and evaluated for cytotoxic and anti–HIV-1 activities.^35^ This illustrates why occurrence-derived novelty should be interpreted alongside conservative evidence integration. Likewise, bioactive lignans reported from *Phyllanthus urinaria* show that compounds belonging to recurrent scaffold families within a lineage should not be excluded from focused follow-up when supported by curated activity evidence.^36^

Additional studies on securinane-type alkaloids from *Flueggea* lineages and dinorditerpenes with anti–hepatitis C virus activity further illustrate that STARS are not limited to a single assay context or chemical class.^37–40^ Collectively, these examples support the interpretation that restriction and scaffold distinctness, when considered together with curated quantitative evidence, identify a compact STARS category for more focused downstream evaluation.

In this context, ChEMBL-linked activity is treated as a conservative evidence layer for candidate stratification, not as independent confirmation. Compounds highlighted here are intended for downstream manual review of assay context and, where relevant, experimental validation.

The HIDDEN GEMS quadrant (Figure 7, Q3) represents a complementary outcome of evidence-aware candidate stratification: lineage-restricted, scaffold-distinct compounds that lack qualifying ChEMBL activity records. Importantly, absence of a ChEMBL record should be interpreted as absence of curated evidence, not absence of bioactivity. Natural-products literature is unevenly represented in standardized bioactivity resources and often emphasizes chemistry-led discovery without target-resolved profiling.^9^

In this sense, the HIDDEN GEMS category captures a conservative evidence gap within the curated chemical space: compounds with strong restriction and scaffold-level novelty that remain sparsely annotated in curated bioactivity databases. Consistent with this interpretation, several Phyllanthaceae-associated chemotypes entered the literature primarily through structural and chemotaxonomic studies, with limited or nonstandardized biological evaluation.

Examples include hydrolyzable tannins, lineage-restricted alkamides, phyllanthimide, podocarpane-type diterpenoids, and the unusual triterpenoid cleistanone.^27–46^ These cases illustrate why scaffold novelty remains informative even when curated activity coverage is incomplete.

Collectively, Figure 7 shows how the compound-centric framework separates the STARS and HIDDEN GEMS categories. The STARS category comprises lineage-restricted compounds with ChEMBL-linked activity records, whereas the HIDDEN GEMS category comprises lineage-restricted compounds that remain sparsely annotated in curated bioactivity resources. The evidence-novelty matrix therefore distinguishes compounds supported by indexed activity evidence from compounds that are chemically distinctive yet still poorly represented in standardized bioactivity databases under the same restriction- and scaffold-aware framework.

The results presented here should be interpreted within the scope of literature-curated and database-indexed chemical knowledge. The primary contribution of this study is the development and demonstration of a reproducible compound-centric framework for organizing reported plant chemical space. It is not intended as an exhaustive reconstruction of Phyllanthaceae metabolism. Phyllanthaceae was used as a chemically diverse case study to illustrate how the workflow can support rationalized searches, pattern visualization, and candidate stratification.

The framework itself is taxon-flexible and can be applied to other families, genera, or species represented in LOTUS. In each case, however, the outputs necessarily reflect the completeness and curation structure of the underlying resources. LOTUS provides a broad and valuable source of species-structure associations, but it does not constitute a complete inventory of all metabolites produced by a lineage. Therefore, the chemical-space maps generated by the workflow should be read as organized views of reported and curated chemistry. They should not be interpreted as direct estimates of the complete metabolome of any plant lineage.

Differences among taxa may reflect biological and chemical variation, but they may also reflect the uneven history of phytochemical investigation. Sampling intensity, analytical accessibility, compound isolation preferences, taxonomic coverage, and reporting practices can all influence apparent richness, class diversity, scaffold recurrence, and the identification of lineage-restricted compounds. WFO reconciliation, InChIKey-based dereplication, Murcko scaffold analysis, and ClassyFire/NPClassifier-derived harmonization reduce several sources of inconsistency. However, they cannot fully eliminate unresolved taxonomic records, incomplete metadata, stereochemical ambiguity, or errors propagated from source publications and databases.

These considerations are also important for interpreting the priority categories. ChEMBL-linked activity records provide a conservative layer of indexed bioactivity evidence, but their absence should be interpreted as lack of qualifying curated evidence under the filters used here, not as absence of biological activity.

Thus, STARS and HIDDEN GEMS should be viewed as evidence-aware, rule-based categories. They support rational candidate stratification and evidence-gap identification rather than definitive biological ranking. Within these boundaries, the framework provides a transparent and reproducible way to mine large-scale occurrence data, visualize lineage-associated chemical patterns, and prioritize compounds for focused expert review and downstream experimental investigation across different taxonomic scales.

## 4 Conclusions

In sum, this work presents a reproducible compound-centric analytical framework for organizing, interpreting, and visualizing plant chemical occurrence data. By integrating WFO-based taxonomic reconciliation, InChIKey-based structure consolidation, Murcko scaffold organization, global family level rarity assessment, physicochemical profiling, and conservative ChEMBL-linked bioactivity evidence, the workflow converts heterogeneous LOTUS-derived records into structure-resolved and evidence-stratified chemical-space maps.

Applied to Phyllanthaceae, the framework revealed genus-level variation in reported chemical repertoires, highlighted distinct physicochemical regimes, distinguished scaffold recurrence from scaffold novelty, and separated lineage-restricted compounds with indexed bioactivity evidence from lineage-restricted compounds that remain poorly represented in curated bioactivity databases. These results show that the framework moves beyond simple occurrence retrieval. It generates auditable outputs for data mining, pattern recognition, evidence-gap identification, and evidence-aware candidate stratification in natural products research. More broadly, its taxon-flexible design enables reproducible analyses across multiple taxonomic scales represented in LOTUS. It also provides a transparent basis for exploring chemical diversity and supporting focused downstream investigation.

## Supplementary Material





## Notes

Hybrid-class composition of the Top 15 Murcko scaffolds in Phyllanthaceae (Figure S1) (PDF)
Supplementary data workbook containing the curated compound-level ranking output, STARS and HIDDEN GEMS subsets, scaffold metrics, genus-level physicochemical summaries, and significant pairwise comparisons used in the compound-centric framework (XLSX)
