# Supplementary material for: A Compound-Centric Framework for Mapping Plant Chemical Space through Structural Scaffolds and Bioactivity Evidence
Source: ACS Omega. 2026 Jun 19;11(26):39350–62. doi: 10.1021/acsomega.6c04145 (PMC13347387; doi:10.1021/acsomega.6c04145)
Supplement: Supplementary file 1 [file ao6c04145_si_001.pdf]

Supporting Information

A Compound-Centric Framework for Mapping Plant Chemical Space through Structural Scaffolds and Bioactivity Evidence

Carollo, Carlos Alexandre*[,1]; Sorrilha-Rodrigues, Andrey Gaspar[1]; Nocetti, Mariana Calarge[1,2]; Alves, Flavio Macedo[3]; Carollo, Aline Regina Hellmann[2].

1 Laboratory of Natural Products and Mass Spectrometry (LaPNEM), Federal University of Mato Grosso do Sul, 79070-900, Campo Grande, MS, Brazil

2 LaSMiNano - Laboratory of Micro- and Nanostructured Systems, Federal University of Mato Grosso do Sul, 79070-900, Campo Grande, MS, Brazil

3 Laboratory of Botany, Federal University of Mato Grosso do Sul, 79070-900, Campo Grande, MS, Brazil

*Corresponding author: Carlos Alexandre Carollo - carlos.carollo@ufms.br

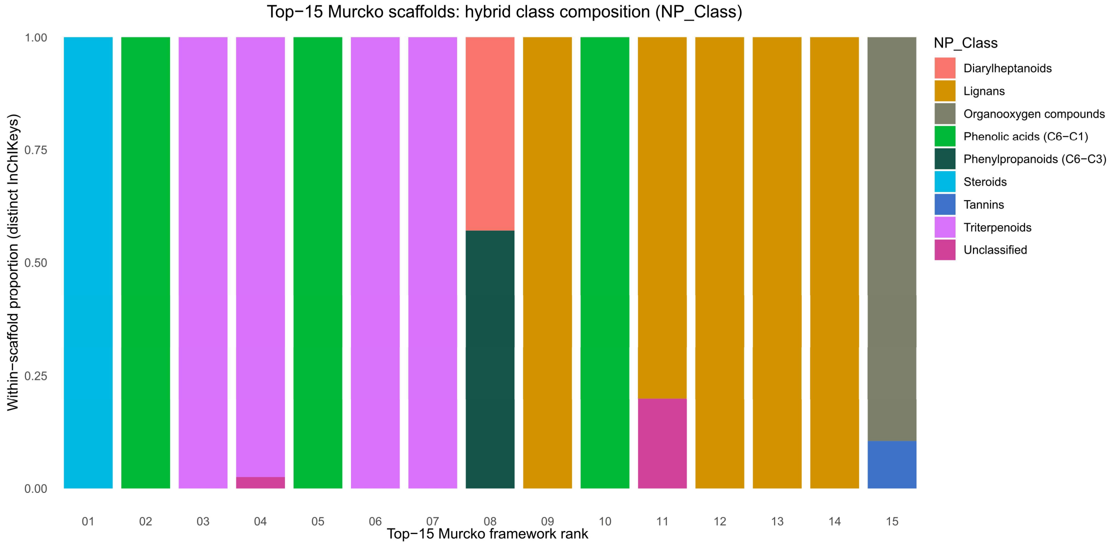

Figure S1. Hybrid-class composition of the Top 15 Murcko scaffolds in Phyllanthaceae. Stacked bars show, for each of the Top 15 Murcko frameworks (fragment MW ≥ 200 Da; ranked by frequency), the within-scaffold proportion of distinct InChIKeys assigned to each hybrid chemical class (NP_Class). Values sum to 1 within each scaffold, allowing direct comparison of class signatures across frameworks independent of total scaffold frequency.
